# Bone Specific Alkaline Phosphatase and Serum Calcification Propensity Are Not Influenced by Etelcalcetide vs. Alfacalcidol Treatment, and Only Bone Specific Alkaline Phosphatase Is Correlated With Fibroblast Growth Factor 23: Sub-Analysis Results of the ETACAR-HD Study

**DOI:** 10.3389/fmed.2022.948177

**Published:** 2022-07-06

**Authors:** Katharina Dörr, Sebastian Hödlmoser, Michael Kammer, Roman Reindl-Schwaighofer, Matthias Lorenz, Bianca Reiskopf, Rahel Jagoditsch, Rodrig Marculescu, Rainer Oberbauer

**Affiliations:** ^1^Department of Nephrology, Medical University of Vienna, Vienna, Austria; ^2^Section for Clinical Biometrics, Center for Medical Statistics, Informatics and Intelligent Systems (CeMSIIS), Medical University of Vienna, Vienna, Austria; ^3^Vienna Dialysis Center, Vienna, Austria; ^4^Department of Laboratory Medicine, Medical University of Vienna, Vienna, Austria

**Keywords:** calcimimetic, vitamin D, calcification, bone turn over, hemodialysis

## Abstract

Secondary hyperparathyroidism in chronic kidney disease poses a major risk factor for vascular calcification and high bone turnover, leading to mineralization defects. The aim was to analyze the effect of active vitamin D and calcimimetic treatment on fibroblast growth factor 23 (FGF23), serum calcification propensity (T50), a surrogate marker of calcification stress and bone specific alkaline phosphatase (BAP) in hemodialysis. This is a subanalysis of a randomized trial comparing etelcalcetide vs. alfacalcidol in 62 hemodialysis patients for 1 year. We compared the change of BAP and serum calcification propensity between the two medications and assessed the influence of FGF23 change over time. We found no significant differences in the change of BAP or serum calcification propensity (T50) levels from baseline to study end between treatment arms (difference in change of marker between treatment with etelcalcetide vs. alfacalcidol: BAP : 2.0 ng/ml [95% CI-1.5,5.4], *p* = 0.3; T50: –15 min [95% CI –49,19], *p* = 0.4). Using FGF23 change over time, we could show that BAP levels at study end were associated with FGF23 change (–0.14 [95% CI –0.21, –0.08], *p* < 0.001). We did not observe the same association between FGF23 change and T50 (effect of FGF23 change on T50: 3.7 [95% CI –5.1, 12], *p* = 0.4; *R*^2^ = 0.07 vs. *R*^2^ = 0.06). No significant difference was found in serum calcification propensity (T50) values between treatment arms. FGF23 was not associated with serum calcification propensity (T50), but was negatively correlated with BAP underlying its role in the bone metabolism.

**Clinical Trial Registration:**

[www.ClinicalTrials.gov], identifier [NCT03182699].

## Introduction

The substantially increased risk of cardiovascular morbidity and mortality in chronic kidney disease (CKD) is promoted by calcification of soft tissues and the vasculature (1). By the time patients reach dialysis, the prevalence and progression of vascular calcification (VC) rises rapidly (2). Secondary hyperparathyroidism (sHPT) is present in up to 80% of patients with end-stage kidney disease (ESKD) and poses a key risk factor for the initiation and development of VC as well as high bone turnover and bone mineral loss (3–5). Calcimimetic therapy is effective in lowering serum concentrations of fibroblast growth factor 23 (FGF23), which is a risk factor for left ventricular hypertrophy (LVH) and cardiovascular events in people with CKD (6, 7). Calcimimetics bind to the parathyroid calcium sensing receptor, causing a leftward shift in the set-point for calcium-regulated parathyroid hormone (PTH) secretion and therefore decrease the PTH concentration and serum calcium levels (8). Yu et al. showed that etelcalcetide (ETL) prevented VC in a rat model with CKD and sHPT (3). ETL can effectively reduce the levels of intact PTH, calcium, phosphate and FGF23 in ESRD patients (9, 10). In contrast, VC was found to be enhanced under the treatment with vitamin D analogs, which frequently led to hypercalcemia, hyperphosphatemia and increased levels of FGF23 (11, 12). Both calcimimetics and vitamin D analogs are common therapeutic options of sHPT.

The serum calcification propensity test (T50) was performed as a surrogate marker of calcification stress and mortality in CKD patients (13, 14). Bundy et al. showed that these patients exhibit a shortened time to serum calcification (15). Furthermore, Shoji et al. reported that treatment with ETL is more effective in increasing the T50 value indicating a lower serum calcification propensity than the selective vitamin D receptor activator maxacalcitol in hemodialysis patients (4).

Another marker of vascular calcification is the activity of bone specific alkaline phosphatase (BAP) (16). Andrukhova et al. from Vienna published that FGF23 suppresses the expression of osteocytic tissue non-specific AP in an autocrine/paracrine manner. This contributes to the mineralization defect, which is one of the hallmarks of CKD-mineral and bone disorder (MBD) (17, 18).

We recently performed a randomized, controlled trial on hemodialysis patients with sHPT, confirming the suppression of FGF23 by calcimimetic treatment with ETL and its increase under alfacalcidol (ALFA) (19). In the present study we sought to evaluate the effect of the study medication on markers of calcification and bone turnover.

## Materials and Methods

### Study Objective, Design, and Participants

The primary objective of the study was to elucidate, whether ETL vs. ALFA treatment differently effect the serum calcium propensity determined by serum calcification propensity test (T50) and the bone turnover marker BAP. In addition, the effect of FGF23 on these two parameters was analyzed.

Data were obtained from the randomized, controlled, single-blinded trial of people on maintenance hemodialysis with sHPT and LVH. Sixty-two patients were randomized in a 1:1 ratio to intravenous ETL or ALFA for 1 year. We enrolled patients who were on dialysis treatment between 3 and 36 months with sHPT. The trial was approved by the ethics committee of the Medical University of Vienna [MUV (EK # 1127/2017)], the national regulatory authorities (AGES # 10087746) and conducted in accordance with the principles of the Declaration of Helsinki. Details on the trial design, including a complete list of inclusion and exclusion criteria were published previously (19–21).

### Laboratory Analysis

Biochemical data were collected prior to the hemodialysis session and before the start of ETL or ALFA treatment. For the serum calcification propensity (T50) analysis two serum samples per patient were collected, one at baseline and one after 52 weeks of treatment. In case of discontinuation of the trial prior to 1 year, the second analysis was performed on the last day of treatment. The samples were stored at –80^°^C and shipped on dry ice to the Calciscon Laboratory in Switzerland where the calcification propensity, i.e., the conversion time from primary to secondary calciprotein particles was quantified (22). A Nephelostar nephelometer was used to detect the time-resolved changes of laser light scattering associated with the transformation.

BAP was measured at 9 timepoints during the trial from frozen serum probes (chemiluminescent immunoassay, DiaSorin^®^, measurement batched, duplicate, CV 7–8%). Intact PTH (electrochemiluminescence immunoassay, Roche^®^, measurement in real time, duplicate, CV < 1.7%), calcium (NM-BAPTA photometric test, Roche, measurement in real time, duplicate, CV ≤ 2%), phosphate (ammonium molybdate UV photometric test, Roche, measurement in real time, duplicate, CV 0.9%), 25-hydroxyvitamin D [25(OH)D] and 1,25-dihydroxyvitamin D [1.25(OH)2D] (chemiluminescent immunoassay, Roche^®^, measurement batched, duplicate, CV 7–9% for 25-OH and 5.9% for 1.25OH) were measured every 2 weeks in the first 10 weeks, followed by measurements every 4 weeks. Serum calcium was corrected for albumin (bromcresol green method CV ≤ 1.8%) (23). Intact FGF23 (chemiluminescent immunoassay, DiaSorin^®^, measurement batched, duplicate, CV ≤ 3.8%). And αKlotho (FluoBolt™, Fianostics, batched, duplicate, CV ≤ 8%) were measured in 8-week-intervals.

### Statistical Analysis

We summarized baseline characteristics of the study cohort using median (IQR) for continuous variables and counts (%) for categorical variables. To visualize biomarker trajectories over time, we depicted boxplots for each treatment arm at baseline and in 12-week increments. For each interval after baseline, the boxplots represent all data in the preceding 12 weeks. We added non-parametric smooth curves (from the “loess” function in *R*) to the boxplots to underline the mean biomarker trajectories over time.

To assess the effect of the treatment (ETL vs. ALFA) on BAP and serum calcification propensity (T50), we used ANCOVA models for each marker to estimate the difference between baseline and study end, adjusted for the respective baseline value and the two stratification factors, i.e., residual kidney function (≥ 500 mL/d vs. < 500 mL/d) and dialysis center (MUV vs. WDZ). To model the effect of FGF23 on BAP and serum calcification propensity (T50), we first fitted a linear mixed effects regression model to estimate the change of FGF23 over time per patient. Due to its skew distribution, FGF23 entered the model on a log_2_ scale. The model included an intercept and slope as fixed effects, as well as a random intercept and slope per patient. We interpreted the sum of the fixed and random slope parameter as FGF23 change per time unit. This approach allowed us to include all available FGF23 measurements to estimate FGF23 change over time per patient. In a second step, we modeled biomarker levels at the end of the study by two linear regression models for BAP and serum calcification propensity (T50), respectively. The first model included FGF23 change and baseline BAP/serum calcification propensity (T50) as covariates, in the second model we excluded FGF23 change. We then compared the parameter estimates as well as explained variance (*R*^2^) of the two approaches to assess the influence of FGF23 change on model fit.

For all models, we conducted distribution and residual analysis to check for model assumptions and possible outliers. Due to some very high outliers, we clipped the BAP and serum calcification propensity (T50) levels at baseline and at study end at the respective 97.5% quantiles. In all analyses, we regarded two-sided *P*-values < 0.05 as significant. Because of the descriptive nature of this subanalysis of a randomized controlled trail, we did not adjust any *P*-values for multiple testing. All statistical analysis was conducted using R 4.0.4.

## Results

### Baseline Characteristics and Biomarker Trajectories

The study cohort included 62 patients from the original trial, 32 of whom were treated with ETL and another 30 with ALFA. Table 1 summarizes the study cohort characteristics at baseline and shows, that the two treatment groups were well balanced at the beginning of the two treatment arms. A detailed description of the study cohort and the original study design are published elsewhere (19, 20).

**TABLE 1:** Study population characteristics at baseline in the two treatment arms, etelcalcetide and vitamin D.

	ETL, *N* = 32	ALFA, *N* = 30
Age	66 (53, 71)	62 (54, 66)
**Sex**		
Female	10 (31%)	6 (20%)
Male	22 (69%)	24 (80%)
BMI	27.9 (24.2, 33.1)	26.5 (23.5, 29.2)
Months on dialysis before baseline	11 (4, 19)	12 (5, 23)
Residual kidney function		
≤500 mL/d	6 (19%)	6 (20%)
> 500 mL/d	26 (81%)	24 (80%)
**Dialysis Center**		
MUV	10 (31%)	10 (33%)
WDZ	22 (69%)	20 (67%)
**Comorbidities**		
Diabetes	14 (44%)	12 (40%)
Hypertension	32 (100%)	29 (97%)
Hyperlipidemia	15 (47%)	14 (47%)
Peripheral vascular disease	7 (22%)	6 (20%)
Coronary artery disease	18 (56%)	20 (67%)
Heart failure	13 (41%)	6 (20%)
**Medication**		
Phosphate binders—screening	26 (81%)	23 (77%)
Phosphate binders—increase	9 (28%)	21 (70%)
Calcium supplementation—screening	1 (3.1%)	2 (6.7%)
Calcium supplementation—increase	4 (12%)	3 (10%)

*Continuous variables are described by median (Q1 Q3), categorical variables by count (%). Age, residual kidney function, comorbidities and medication at screening were determined at the initial screening visit before randomization; Phosphate binders include Sevelamer hydrochloride, Lathanum carbonate, Aluminiumchloride-hydroxide, Ferric citrate hydrate. Calcium supplementation includes Calcium acetate, Calcium chloride. Medication increase is defined as an elevation of the dosage or the addition of another drug to the preexisting phosphate binder or calcium supplement. MUV, Medical University of Vienna; WDZ, Vienna Dialysis Center; ETL, Etelcalcetide; ALFA, Alfacalcidol.*

**FIGURE 1:**
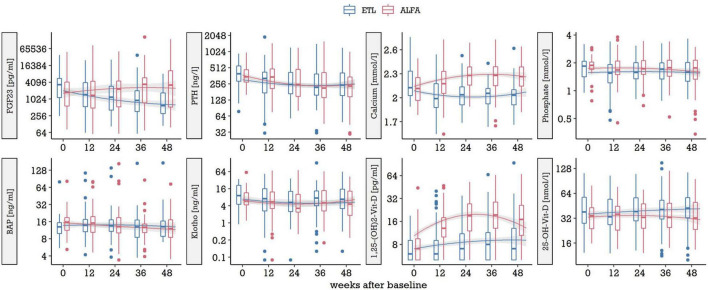
Biomarker levels at baseline and over time in the two treatment arms, ETL and ALFA. The boxplots in 12-week-increments represent all data in the preceding 12 weeks, by treatment, on a log_2_ scale. Trajectories are represented by non-parametric smoothing (loess curves). ETL, Etelcalcetide; ALFA, Alfacalcidol.

The serum levels of FGF23 (pg/ml), iPTH (ng/l), calcium (mmol/l), phosphate (mmol/l), BAP (ng/ml), klotho (ng/ml), 1.25-OH_2_-Vitamin D (pg/ml), and 25-OH-Vitamin D (nmol/l) at baseline and over time is provided in Figure 1. Mean baseline levels were similar for all biomarkers. Trajectories of 1.25-OH_2_-Vitamin D, calcium and FGF23 diverged over time in the two treatment groups, while the other markers did show a similar course in the ETL and ALFA group. Ten patients dropped out of the trial before completing 1 year of treatment, five in each treatment arm. Laboratory analyses were performed using probes from all 62 patients. In case of drop-out, the last collected probe was used for serum calcification propensity (T50) measurement.

### Effect of Etelcalcetide and Its Increase Under Alfacalcidol Treatment on Bone Specific Alkaline Phosphatase and Serum Calcification Propensity

**FIGURE 2:**
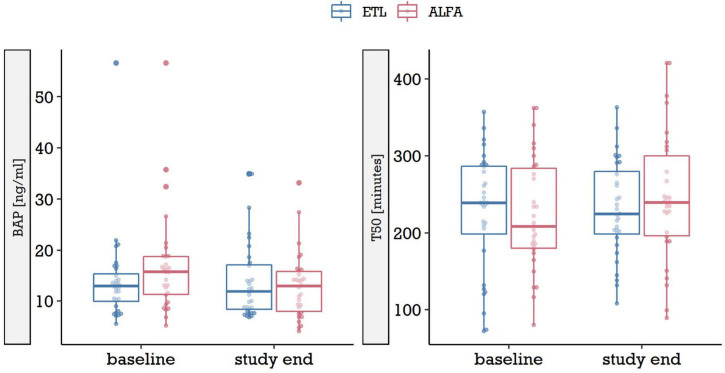
BAP and serum calcification propensity score test (T50) at baseline and study end in the two treatment arms, ETL and ALFA. Due to outliers with very high levels, for each marker and point in time the data was clipped at the respective 97.5% quantile, i.e., levels above it were set to the 97.5% quantile [3 observations in BAP, 4 in serum calcification propensity score test (T50)]. ETL, Etelcalcetide; ALFA, Alfacalcidol.

We found no significant differences in the change of BAP or serum calcification propensity (T50) with respect to the two treatment arms, ETL and ALFA. Figure 2 compares BAP and serum calcification propensity (T50) at baseline and at study end for both medications, after clipping very high outliers at the 97.5% quantile (3 observations of BAP, 4 observations of T50). Median [IQR] change in BAP throughout the study period was 0.05 [–3.17, 2.75] under ETL treatment and –1.85 [–6.15, –0.02] in the ALFA group. In serum calcification propensity (T50), median change amounted to –1.5 [–59.25, 56.25] in ETL treatment and 19.9 [–32.25, 77.25] in ALFA treatment. We assessed differences between baseline and study end in ANCOVA models, in which we modeled the adjusted change in marker levels per patient. The models were summarized in Table 2. Both ANCOVA models showed that after controlling for baseline value and randomization factors, there were no significant differences in BAP and serum calcification propensity (T50) change between the treatment arms (estimated difference in change of BAP, ETL vs. ALFA: 2.0 ng/ml [95% CI –1.5, 5.4], *p* = 0.3; estimated difference in T50, ETL vs. ALFA: –15 min [95% CI –49, 19], *p* = 0.4).

**TABLE 2:** ANCOVA models for change in BAP and serum calcification propensity score test (T50) from baseline to study end.

	Change in BAP		Change in T50	
	Beta (95% CI)	*P*-value	Beta (95% CI)	*P*-value
Medication ETL vs. ALFA	2.0 (–1.5, 5.4)	0.3	–15 (–49, 19)	0.4
Baseline value (BAP / T50)	–0.68 (–0.86, –0.50)	<0.001	–0.74 (–1.0, –0.5)	<0.001
Residual kidney function > 500 mL/d vs. ≤ 500 mL/d	–1.8 (–6.6, 2.9)	0.4	4.3 (–44, 53)	0.9
Dialysis center WDZ vs. MUV	1.3 (–2.8, 5.4)	0.5	–62 (–102, –21)	0.004

*Models were adjusted for baseline BAP and T50 values, and the randomization variables of the original trial (residual kidney function and dialysis center). ETL, Etelcalcetide; ALFA, Alfacalcidol; MUV, Medical University of Vienna; WDZ, Vienna Dialysis Center.*

### Association of Fibroblast Growth Factor 23 Change With Bone Specific Alkaline Phosphatase and Serum Calcification Propensity

We found indications that FGF23 change over time was associated with BAP at study end, but not with serum calcification propensity (T50). The linear mixed model to assess FGF23 change per patient delivered a marginal R squared of $R_{m}^{2}=0.008$ and a conditional R squared of $R_{c}^{2}=0.87.$ The latter seems, in comparison to ordinary linear regression, rather high. However, for a mixed effects model, $R_{c}^{2}$ denotes the explained variance when the (population level) fixed effects as well as the (individual level) random effects are taken into account. This flexibility per individual contributes to the high goodness-of-fit. While a high $R_{c}^{2}$is of limited value for prediction purposes, in the context of this study, were we used the mixed model to describe a trait per individual, it shows that the approach is adequate to assess FGF23 change per patient.

In Table 3, we summarized the models for BAP and serum calcification propensity (T50) at study end with and without FGF23 change as covariate. Again, we clipped the baseline and study end levels at the respective 97.5% quantiles. We could show that the estimated effect of FGF23 change on BAP at study end was significant (–0.14 [95% CI –0.21, –0.08], *p* < 0.001) and that the inclusion of FGF23 change into the model substantially increased model fit (*R*^2^ = 0.27 for model without, and *R*^2^ = 0.46 for model with FGF23 change as covariate). Interpretation of these regression coefficients is not straight forward, since the outcome (BAP) was modeled on a log_2_ scale. When transformed to the original scale, the additive regression coefficients turn into multiplicative factors. This means that for every unit step of FGF23 change [which denotes the change of log_2_(FGF23)], BAP levels are multiplied (or divided, when the covariate is reduced) by a factor of 2^–0.14^ = 0.91. We found no indication for an effect of FGF23 change on serum calcification propensity test (T50), as neither the regression coefficient was significant, nor did the model performance decrease when FGF23 change was excluded (effect of FGF23 change on T50: 3.7 [95% CI –5.1, 12], *p* = 0.4; *R*^2^ = 0.06 in the full model vs. *R*^2^ = 0.07 when FGF23 was excluded).

**TABLE 3:** Linear regression models for BAP and serum calcification propensity score test (T50) at study end.

		Full model		Excluding FGF23 change	
Dependent variable	Covariate	Beta (95% CI)	*P*-value	Beta (95% CI)	*P*-value
BAP at study end	FGF23 change	–0.14 (–0.21, –0.08)	<0.001		
	Baseline BAP (log_2_)	0.56 (0.36, 0.76)	<0.001	0.54 (0.31, 0.77)	<0.001
	*R* ^2^	0.46		0.27	
T50 at study end	FGF23 change	3.7 (–5.1, 12)	0.4		
	Baseline T50	0.23 (–0.01, 0.48)	0.063	0.24 (–0.01, 0.49)	0.056
	*R* ^2^	0.07		0.06	

*Models include the respective baseline level of the marker and either include (“Full model”) or exclude FGF23 change over time per patient as covariate. Baseline BAP entered the model on a log_2_ scale. FGF23 change per patient was estimated in a linear mixed effects regression model. Explained variance, denoted by R^2^, is compared between the models to assess the effect of FGF23 change over time on model fit.*

## Discussion

In this trial the effects of ETL and ALFA on serum calcification propensity test (T50) and BAP were compared in hemodialysis patients with sHPT. In addition, the association of FGF23 with these markers was analyzed. We found no significant differences between change in T50 between medication groups after 1 year of treatment. Mean T50 increased under ALFA while it decreased under ETL, which stands in contrasts to the results by Shoji et al.

The change in FGF23, i.e., its decrease under ETL and increase under ALFA, was also not associated with serum calcification propensity (T50). In Shoji’s trial both ETL and vitamin D increased T50, while its increase was more effective under ETL. The authors explained this between-group difference by the different effects of the drugs on calcium and phosphate levels, which serum calcification propensity test (T50) itself was reported to be inversely correlated with in the past (24). In our trial, serum calcium levels also decreased under calcimimetic treatment and increased under ALFA therapy. However, we showed similar courses of phosphate levels in both treatment groups. As reported before, this unexpected finding could be explained by an increased use of phosphate binder therapy in the ALFA group in our study. In Shoji’s trial on the other hand, phosphate trajectories were lower in the ETL group. In addition, even though the proportion of patients under phosphate binder therapy was comparable between groups at baseline, the graph showing the changes in the medications during the course of the trial indicate a slightly higher use of phosphate binders in the ETL group, possibly explaining the differences between trials in phosphate levels under the same therapy. It is also important to point out, that the serum calcification propensity values in Shoji’s trial were much lower than those reported for CKD patients from western countries. The explanation for this occurrence remains unclear. In addition, the trial used different target ranges of sHPT parameters. Especially in the case of iPTH, the target range was 60–240 ng/l as recommended by the clinical practice guideline for the management of CKD-MBD by the Japanese Society for Dialysis Therapy and patients were included when iPTH was above 240 ng/l, which is lower in comparison to our trial (inclusion if iPTH > 300 ng/l, target range between 100 and 300 ng/l). Furthermore, concomitant therapy differed, allowing for oral calcimimetic use in the vitamin D group, which was prohibited in our trial. As the authors correctly point out, the results of the Japanese study may not be applicable to patients in other countries.

In the present study, we showed an inverse association of FGF23 with BAP. So far, the isoenzyme BAP, which comprises approximately 50% of total circulating alkaline phosphatase (AP) is mostly known as a bone turnover marker in CKD-MBD patients reflecting the bone formation in skeletal tissue (25–27). High levels of BAP were shown to be strongly associated with mortality in dialysis patients (28). In animal models FGF23 suppresses tissue non-specific alkaline phosphatase (TNAP) transcription and also phosphate production in osteoblastic cells through FGF receptor-3 independent of klotho, leading to a bone mineralization defect in an autocrine/paracrine manner (29, 30). A trial by Slouma et al. showed that both FGF23 and BAP can be used as markers of bone fragility in hemodialysis patients (31). Regarding VC, higher BAP was significantly associated with its presence in hand arteries in a trial with 167 hemodialysis patients (32). It was also positively correlated with abdominal aortic calcification in the same patient collective (33). Total AP was shown to be lowered under oral calcimimetic in the past, but BAP was not assessed in that analysis in hemodialysis patients (34).

There are some limitations in our study. First, this is a subanalysis of a randomized controlled trial, powered to compare the effects of ETL and ALFA on LVH in hemodialysis, not to detect a difference of the study medication or FGF23 on calcification and bone turnover markers. Second, it also did not compare the effects of the study drugs on clinical hard end points but rather on surrogate biomarkers of bone and mineral disorders. Trial participants were also not regularly investigated by skeletal imaging. Third, considering that the per protocol analysis of the trial by Shoji et al. included a much higher number of participants (*n* = 319), our trial had a comparatively low sample size.

The strengths of the trial, however, include its prospective, randomized design, a completed follow-up and the use of intravenously administered drugs, avoiding the issue of non-adherence, which is known to be very high especially for calcimimetics in hemodialysis patients (35). In addition, the T50 test which was used in this trial was previously shown to accurately assess the formation rate of calciprotein particles and therefore reflect the calcification risk in ESKD and was also shown to be associated with mortality in this collective (24).

In conclusion, this study showed that treatment with ETL was not effective in decreasing the serum calcification propensity in comparison with ALFA and unrelated to BAP change. FGF23 was associated with BAP, but not with serum calcification propensity (T50). The translation of our findings into clinical end points necessitates a different trial setting with a much higher number of patients and a longer follow-up period. From our experience, the use of calcimimetics in hemodialysis patients is a safe, effective and well tolerated option for the treatment of sHPT. We would recommend the use of intravenous calcimimetics and close monitoring of calcium levels.

## Data Availability Statement

The raw data supporting the conclusions of this article will be made available by the authors, without undue reservation.

## Ethics Statement

The studies involving human participants were reviewed and approved by Ethics Committee of the Medical University of Vienna [MUV (EK # 1127/2017)]. The patients/participants provided their written informed consent to participate in this study.

## Author Contributions

KD, RO, RR-S, and SH were responsible for the conceptualization and methodology of the trial. KD was tasked with the investigation, including data collection, with support by ML, BR, and RJ. SH and MK were assigned with the formal analysis and software. RM was responsible for laboratory analysis. Writing of the original draft was performed by KD and reviewed and edited by the co-authors. All authors contributed to the article and approved the submitted version.

## Conflict of Interest

RO reports grants from Amgen during the conduct of the study. In addition, RO, KD, and RR-S have a patent “Methods of treating left ventricle hypertrophy” pending. The remaining authors declare that the research was conducted in the absence of any commercial or financial relationships that could be construed as a potential conflict of interest.

## Publisher’s Note

All claims expressed in this article are solely those of the authors and do not necessarily represent those of their affiliated organizations, or those of the publisher, the editors and the reviewers. Any product that may be evaluated in this article, or claim that may be made by its manufacturer, is not guaranteed or endorsed by the publisher.

## Abbreviations

AP: alkaline phosphatase
BAP: bone specific alkaline phosphatase
CKD: chronic kidney disease
CKD-MBD: chronic kidney disease – mineral and bone disorder
ESKD: end-stage kidney disease
EtECAR-HD: Effect of etelcalcetide on cardiac hypertrophy in hemodialysis patients–a randomized controlled trial
ETL: etelcalcetide
FGF23: fibroblast growth factor 23
LVH: left ventricular hypertrophy
MUV: Medical University of Vienna
iPTH: intact parathyroid hormone
sHPT: secondary hyperparathyroidism
VC: vascular calcification
WDZ: Vienna dialysis center.
